# Mechanosensitive ion channel gene survey suggests potential roles in primary open angle glaucoma

**DOI:** 10.1038/s41598-023-43072-3

**Published:** 2023-09-23

**Authors:** Wendy W. Liu, Tyler G. Kinzy, Jessica N. Cooke Bailey, Zihe Xu, Pirro Hysi, Janey L. Wiggs, NEIGHBORHOOD Consortium, R. Rand Allingham, Murray Brilliant, Donald L. Budenz, John H. Fingert, Douglas Gaasterland, Teresa Gaasterland, Jonathan L. Haines, Michael A. Hauser, Richard K. Lee, Paul R. Lichter, Yutao Liu, Syoko Moroi, Jonathan Myers, Louis R. Pasquale, Margaret Pericak-Vance, Anthony Realini, Doug Rhee, Julia E. Richards, Robert Ritch, Joel S. Schuman, William K. Scott, Kuldev Singh, Arthur J. Sit, Douglas Vollrath, Robert N. Weinreb, Gadi Wollstein, Donald J. Zack

**Affiliations:** 1 Spencer Center for Vision Research, Byers Eye Institute, Stanford University School of Medicine, 2370 Watson Court, Palo Alto, CA 94303 USA; 2 Department of Population and Quantitative Health Sciences, Cleveland Institute for Computational Biology, Case Western Reserve University, Cleveland, OH USA; 3 Department of Ophthalmology, King’s College London, St. Thomas’ Hospital, London, UK; 4 Department of Twin Research and Genetic Epidemiology, King’s College London, St. Thomas’ Hospital, London, UK; 5 Massachusetts Eye and Ear, Harvard Medical School Boston, Boston, MA USA; 6 Department of Ophthalmology, Duke University Medical Center, Durham, NC USA; 7 Center for Human Genetics, Marshfield Clinic Research Foundation, Marshfield, WI USA; 8 Department of Ophthalmology, University of North Carolina, Chapel Hill, NC USA; 9 Department of Ophthalmology, College of Medicine, University of Iowa, Iowa City, IA USA; 10 Eye Doctors of Washington, Chevy Chase, MD USA; 11 Scripps Genome Center, University of California at San Diego, San Diego, CA USA; 12 Departments of Medicine and Ophthalmology, Duke University, Durham, NC USA; 13 Bascom Palmer Eye Institute, University of Miami Miller School of Medicine, Miami, FL USA; 14 Department of Ophthalmology and Visual Sciences, University of Michigan, Ann Arbor, MI USA; 15 Department of Cellular Biology and Anatomy, Georgia Regents University, Augusta, GA USA; 16 James and Jean Culver Vision Discovery Institute, Georgia Regents University, Augusta, GA USA; 17 Wills Eye Hospital, Philadelphia, PA USA; 18 Department of Ophthalmology, Icahn School of Medicine at Mount Sinai, New York, NY 10029 USA; 19 Institute for Human Genomics, University of Miami Miller School of Medicine, Miami, FL USA; 20 Department of Ophthalmology, West Virginia University Eye Institute, Morgantown, WV USA; 21 Department of Ophthalmology, Case Western Reserve University School of Medicine, Cleveland, OH USA; 22 Department of Ophthalmology, Einhorn Clinical Research Center, New York Eye and Ear Infirmary of Mount Sinai, New York, NY USA; 23 Department of Ophthalmology, NYU School of Medicine, New York, NY USA; 24 Department of Ophthalmology, Mayo Clinic, Rochester, MN USA; 25 Department of Genetics, Stanford University School of Medicine, Palo Alto, CA USA; 26 Hamilton Glaucoma Center, Shiley Eye Institute, University of California, San Diego, CA USA; 27 Wilmer Eye Institute, Johns Hopkins University Hospital, Baltimore, MD USA

**Keywords:** Disease genetics, Ion channels in the nervous system

## Abstract

Although glaucoma is a disease modulated by eye pressure, the mechanisms of pressure sensing in the eye are not well understood. Here, we investigated associations between mechanosensitive ion channel gene variants and primary open-angle glaucoma (POAG). Common (minor allele frequency > 5%) single nucleotide polymorphisms located within the genomic regions of 20 mechanosensitive ion channel genes in the K2P, TMEM63, PIEZO and TRP channel families were assessed using genotype data from the NEIGHBORHOOD consortium of 3853 cases and 33,480 controls. Rare (minor allele frequency < 1%) coding variants were assessed using exome array genotyping data for 2606 cases and 2606 controls. Association with POAG was analyzed using logistic regression adjusting for age and sex. Two rare *PIEZO1* coding variants with protective effects were identified in the NEIGHBOR dataset: R1527H, (OR 0.17, *P* = 0.0018) and a variant that alters a canonical splice donor site, g.16-88737727-C-G Hg38 (OR 0.38, *P* = 0.02). Both variants showed similar effects in the UK Biobank and the R1527H also in the FinnGen database. Several common variants also reached study-specific thresholds for association in the NEIGHBORHOOD dataset. These results identify novel variants in several mechanosensitive channel genes that show associations with POAG, suggesting that these channels may be potential therapeutic targets.

## Introduction

Primary open-angle glaucoma (POAG) is a progressive, neurodegenerative disease leading to loss of retinal ganglion cells (RGCs) and irreversible blindness^1^. Intraocular pressure (IOP) is the only modifiable risk factor, and is the target of current medical and surgical approaches for glaucoma treatment^1^. Despite the known importance of IOP in disease progression, we only have limited understanding of the proteins and molecules that regulate IOP, and the mechanisms by which elevated IOP leads to RGC loss. As the primary sensors that mediate many responses to mechanical signals^2^, mechanosensitive ion channels may play a role in both IOP regulation and the pathologic responses of RGCs to IOP.

In recent years, significant advances have been made in our understanding of mechanosensitive ion channels, including the discovery of the PIEZO family of mechanically activated channels^3,4^, and the validation of the TRAAK/TREK two-pore domain K + (K2P) channels and TMEM63 (hyperosmolality-gated calcium-permeable) channel families as mechanosensitive^5,6^. In addition, many members of the TRP (transient receptor potential) channel family have been implicated in sensing pressure and stretch in mammals^7^, including TRPV1^8,9^, TRPV2^10^, TRPV4^11^, TRPC1^12,13^, TRPC3^14^, TRPC5^15^, TRPC6^16,17^, TRPA1^18,19^, TRPP1^20^, TRPM3^21^, TRPM4^22^ and TRPM7^23^. Mechanosensitive channel families play diverse physiological roles in human health and disease, ranging from touch, blood-pressure sensing and hearing^4,24–28^. However, their roles in the eye are not entirely clear. Several in vitro and mouse studies have suggested that mechanosensitive ion channels may play a role in glaucoma, either by regulating aqueous humor outflow and IOP^29–37^, or by modulating RGC survival^38–41^. However, clinical evidence in humans implicating the role of mechanosensitive channels in glaucoma is lacking.

In this study, we surveyed mechanosensitive ion channel genes in the K2P, TMEM63, PIEZO and TRP channel families and asked whether common and rare genetic risk variants in these genes are associated with POAG using data from independent human genetic datasets.

## Results

### Association results for common variants

In this study we investigated association results for all single nucleotide polymorphisms (SNPs) with minor allele frequencies (MAF) greater than 5% in individuals with European Ancestry located within the genomic regions harboring the genes of interest including 50 Kb upstream and downstream of the gene to capture regulatory elements. Table 1 lists the SNPs corresponding to each genomic region with the best association result for POAG overall and also for the high-tension glaucoma (HTG) and normal-tension glaucoma (NTG) subgroups. Association results for 4 SNPs reached the significance threshold for POAG overall (rs2841593, *KCNK2* (*TREK1*); rs8016340, *TMEM63C*; rs3124515, *TRPM3*; and rs34419652, *TRPV2*). The *TRPV2* SNP is an expression quantitative trait loci (eQTL) in at least one tissue type in the Gene-Tissue Expression (GTEx) database, suggesting that this SNP can impact gene expression. Two SNPs reached the significance threshold for HTG (rs112014893, *PIEZO2*; rs4738210, *TRPA1*) and 4 SNPs for NTG (rs12709104, *PIEZO1*; rs2026109, *TRPM3*; rs9901098, *TRPV2*; rs12423752, *TRPV4*). We next investigated association for these SNPs and glaucoma phenotypes in FinnGen. While none of these SNPs reached nominal (*P* < 0.05) significance for disease association, two SNPs showed consistent direction of effects including rs34419652 (*TRPV2* and POAG) and rs2026109 (*TRPM3* and NTG) (Supplemental Table 1).

**Table 1:** Association results for common variants in NEIGHBHORHOOD.

	Primary open-angle glaucoma					High-tension glaucoma					Normal-tension glaucoma				
	SNP	Effect allele	Effect	P	eQTL	SNP	Effect allele	Effect	P	eQTL	SNP	Effect allele	Effect	P	eQTL
KCNK2/ TREK1	**rs2841593**	**A**	**0.15**	**0.0024**	**N**	rs1981211	T	− 0.11	0.03	NA	rs2601631	C	− 0.23	0.014	Y
KCNK4/ TRAAK	rs2001003	A	− 0.11	0.01	N	rs12793347	A	− 0.16	0.01	N	rs589030	C	0.14	0.044	N
KCNK10/ TREK2	rs9671728	A	0.14	0.006	NA	rs742887	A	− 0.14	0.012	N	rs9671728	A	0.31	0.004	N
PIEZO1	rs11076707	T	0.14	0.016	NA	rs139846483	A	− 0.18	0.019	NA	**rs12709104**	**A**	− **0.37**	**0.0024**	**Y**
PIEZO2	rs575625	A	0.16	0.004	N	**rs112014893**	**A**	**0.29**	**0.00054**	**N**	rs2584737	A	0.2	0.004	N
TMEM63A	rs12405730	T	0.21	0.006	N	rs12405730	T	0.24	0.015	N	rs113651206	A	0.15	0.04	NA
TMEM63B	rs7763358	T	0.15	0.036	N	rs7765579	A	− 0.22	0.005	Y	rs12663599	T	− 0.19	0.019	Y
TMEM63C	**rs8016340**	**A**	− **0.11**	**0.0023**	**N**	rs8016340	A	− 0.13	0.003	N	rs8016340	A	− 0.16	0.018	N
TRPC1	rs79858942	T	− 0.19	0.013	N	rs79858942	T	− 0.22	0.013	N	rs4259003	A	0.2	0.018	Y
TRPC3	rs10518289	C	0.09	0.008	N	rs34574691	T	0.14	0.009	N	rs1465038	C	− 0.19	0.023	N
TRPC5	NA														
TRPC6	rs34840175	T	− 0.13	0.02	N	rs12785434	T	− 0.21	0.005	N	rs12807799	T	0.27	0.056	N
TRPM3	**rs3124515**	**A**	− **0.12**	**0.0013**	**NA**	rs11142681	A	− 0.44	0.006	N	**rs2026109**	**A**	**0.62**	**0.001**	**N**
TRPM4	rs1716267	A	− 0.22	0.021	NA	rs12461216	C	0.23	0.005	Y	rs2232003	T	0.21	0.013	Y
TRPM7	rs9806676	A	0.43	0.016	N	rs8023464	A	0.39	0.04	N	rs12902819	A	0.29	0.012	N
TRPV1	rs161386	A	− 0.07	0.044	Y	rs12945340	A	− 0.18	0.007	N	rs224503	T	0.19	0.004	N
TRPV2	**rs34419652**	**A**	− **0.13**	**0.0009**	**Y**	rs34419652	A	− 0.15	0.005	Y	**rs9901098**	**A**	− **0.33**	**0.0024**	**Y**
TRPV4	rs4766641	A	0.08	0.014	Y	rs4766641	A	0.11	0.018	Y	**rs12423752**	**T**	− **0.28**	**0.0006**	**N**
TRPA1	rs2587561	A	− 0.12	0.014	N	**rs4738210**	**T**	**0.275**	**0.0001**	**N**	rs7844555	A	− 0.2	0.015	N
PKD2/ TRPP1	rs7696304	A	0.06	0.06	Y	rs2853744	T	− 0.24	0.015	N	rs62308636	T	− 0.16	0.19	Y

*SNP* single nucleotide polymorphism, *eQTL* expression quantitative trait loci.

Significant values are in [bold].

### Association results for exome array variants

DNA variants impacting protein structure (coding variants), while often rare in populations, can have large effects on human traits and disease. To evaluate potential roles for protein coding variants in the mechanosensitive genes in POAG, we evaluated rare (MAF < 1%) variants effecting protein structure (missense, stop-loss and splice-sites) using exome array data available for two of the NEIGHBORHOOD cohorts, NEIGHBOR and MEE (total 2606 cases and 2606 controls).

We first evaluated gene-based association using SKAT-O and did not identify significant association with any of the selected mechanosensitive genes that met the criteria of having at least 3 variants available for analysis (Supplemental Table 2). Interestingly one gene, *TRPC1*, has interesting evidence of association (*P* = 6.34E−3) with POAG in the UK Biobank (defined as ICD-10 code H40, N = 11,588 cases and 383,253 controls) using SKAT-O and only missense alleles (Supplemental Table 2).

**Table 2:** Exome array variants with significant association in NEIGHBHOR and MEE.

Gene	rsID	Variant ID	Protein	CADD score	NEIGHBORHOOD				FinnGen		UKBB	
					MAF case	MAF control	OR	P	OR	P	OR	P
PIEZO1	rs199524784	16-88737727-C-G	splice donor	22.9	7.17E−04	2.40E−03	0.38	0.022	NA	NA	0.89	0.696
PIEZO1	rs148870219	16-88722925-C-T	R1527H	17.8	5.30E−04	2.90E−03	0.18	0.0018	0.89	0.05	0.86	0.327
TMEM63B	rs4714759	6-44147432-G-A	V307M	21.7	0.1146	0.1043	1.13	0.041	1.01E+00	0.73	1.01E+00	0.931
TRPA1	rs61758122	8-72069102-G-A	A122V	25.0	1.76E−03	4.68E−04	4.30	0.024	NA	NA	− 0.326	0.576

*CADD* combined annotation dependent depletion, *MAF* minor allele frequency, *OR* odds ratio.

**Figure 1:**
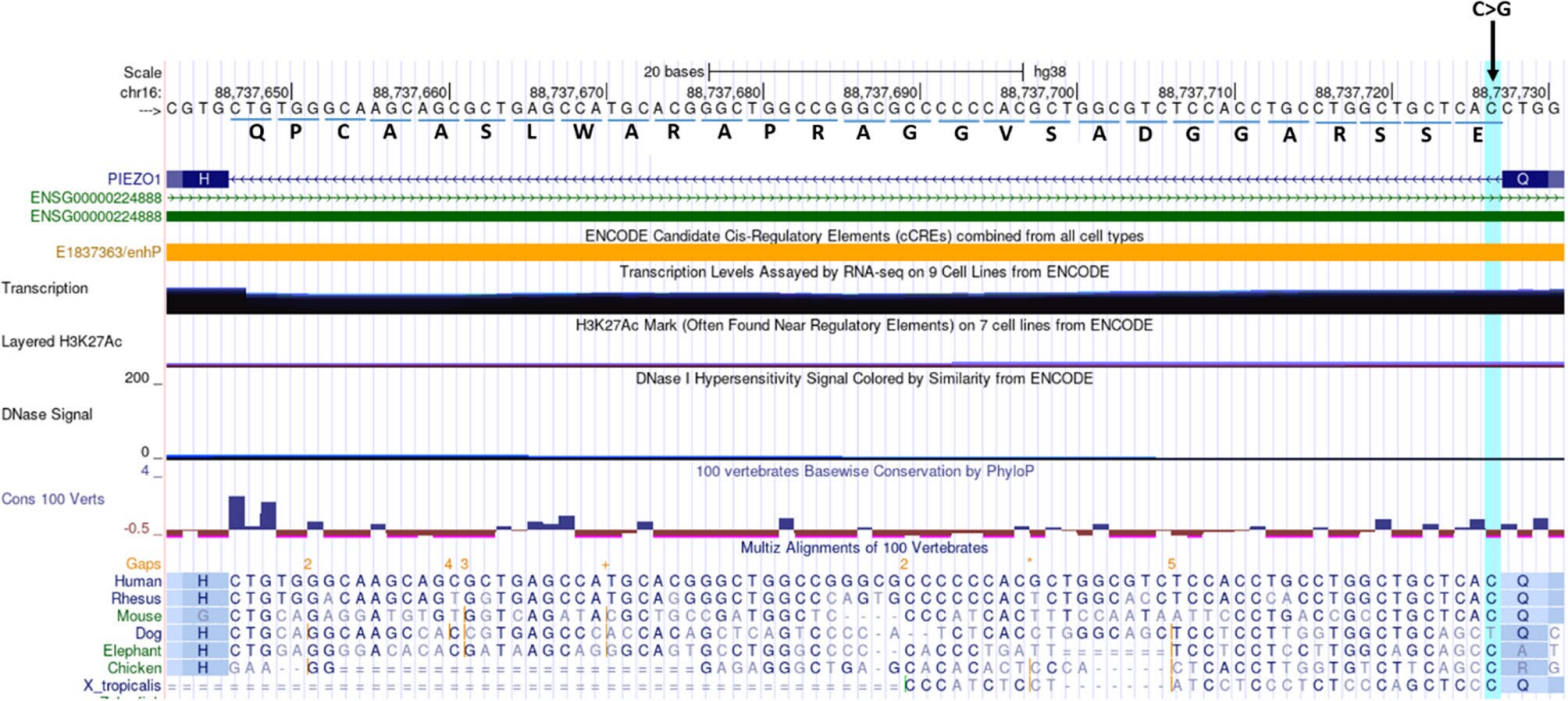
Consequence of *PIEZO1* canonical donor splice variant (16-88737727-C-G). The C > G change disrupts the canonical donor splice sequence GT (the minus strand is shown in the image), leading to retention of the short intron (81 base pairs). A stop codon is not encountered in the reading frame, and as a result 27 amino acids are included in the polypeptide sequence. The amino acids potentially included in the protein are listed below the sequence at the top of the image. Image is rendered from the UCSC genome browser (https://genome.ucsc.edu/).

In the single-variant analysis, 55 variants met the inclusion criteria (MAF < 1%, CADD > 15) (Supplemental Table 3). Two of these variants, both in *PIEZO1*, demonstrated interesting association with POAG in the NEIGHBOR and MEE samples. Both *PIEZO1* variants were found more commonly in controls than in cases suggesting protective effects. The first of these is a missense allele, R1527H (OR 0.17, *P* = 0.0018). This allele is enriched in the Finnish population and the direction of effect was consistent in FinnGen (beta − 0.11, *P* = 0.05) and also in the UK Biobank (beta − 0.149, *P* = 0.347) (Table 2). The second *PIEZO1* allele alters a canonical splice donor site (genomic location 16-88737727-C-G Hg38). The distribution of this variant also suggests protective effects in POAG (OR 0.38, *P* = 0.02) and the direction of effect was also consistent in the UK Biobank (beta = − 0.1, *P* = 0.696) (Table 2). This variant was not present in the FinnGen dataset. The splice variant is predicted to alter splicing (Splice AI = 0.970 (donor_loss)), and incorrect splicing is likely to cause the inclusion of 27 amino acids due to retention of the short intron number 9 (of 50 in the gene) (Fig. 1).

In addition to the *PIEZO1* variants, two other coding variants of interest were identified. The first is a low frequency variant in *TMEM63B* (V307M) that showed nominal association with POAG in the NEIGHBOR and MEE samples (OR = 1.13, *P* = 0.04). This variant also showed consistent evidence of association with POAG in the UK Biobank and also in FinnGen (Table 2). A missense allele in *TRPA1* (A122V) was also nominally associated with POAG in the NEIGHBOR and MEE samples (OR 4.3, *P* = 0.02). However, this variant had the opposite direction of effect in the UK Biobank (beta − 0.326, *P* = 0.58) (Table 2). This variant was not present in the FinnGen dataset.

### Single-cell and single-nucleus RNA sequence data

**Figure 2:**
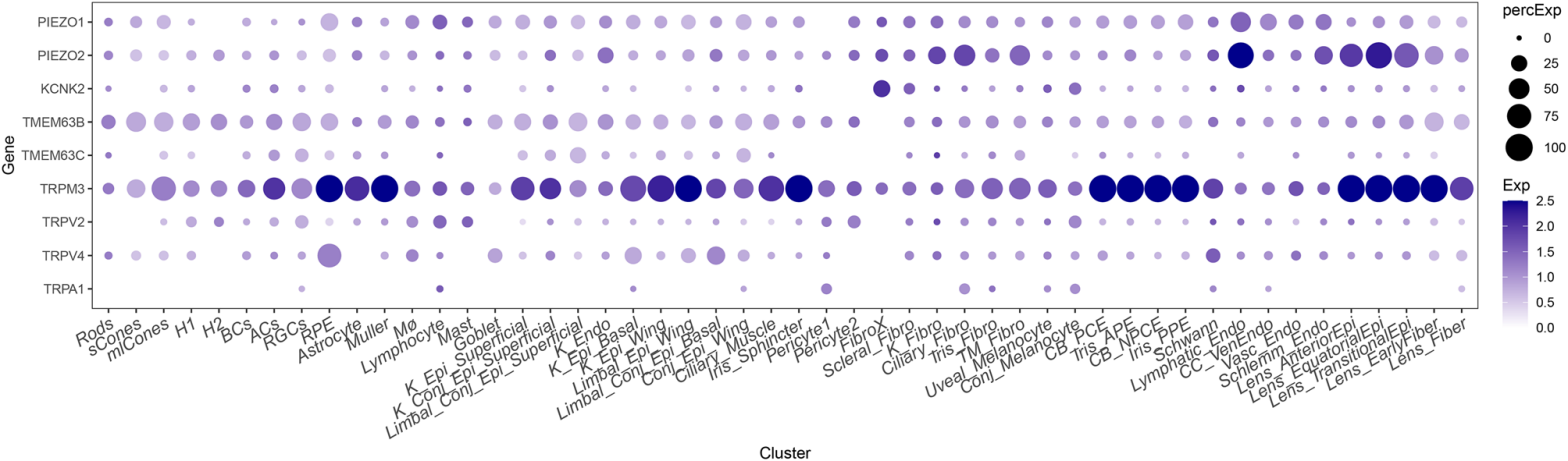
Expression of selected genes with interesting associations with POAG in ocular tissues. Single-cell^43^ and single-nucleus^42^ sequencing data were extracted for anterior segment cell types, neuronal cell types in the retina including retinal ganglion cells, and non-neuronal retinal cell types. Dot plots show expression of selected genes in anterior segment and retinal cell types. Dot size indicates fraction of expressing cells, and color indicates mean expression in expressing cells.

Expression of the mechanosensitive genes in ocular tissues was determined using publicly available single-cell and single-nucleus RNA sequence data^42,43^ (Fig. 2). *PIEZO1* and *PIEZO2* have relatively strong expression in lymphatic endothelial cells in the ocular anterior segment, tissue related to Schlemm’s canal endothelia^42^. *TMEM63B*, *TMEM63C*, *TRPM3* and *TRPV2* all have relatively strong expression in retinal ganglion cells.

## Discussion

Although mechanosensitive channels have been implicated in glaucoma pathogenesis based on experimental studies, the association between mechanosensitive channels genetic variants and glaucoma risk has not been well studied. Here, we surveyed common and rare genetic variants for 20 mechanosensitive channel genes in the K2P, TMEM63, PIEZO and TRP channel families for association with POAG.

Two rare *PIEZO1* coding variants with consistent protective effects for POAG were identified: a missense allele (R1527H) and a variant that alters a canonical splice donor site (genomic location 16-88737727-C-G Hg38). These variants also showed similar effects in the UK Biobank and the R1527H also in the FinnGen database. A *PIEZO1* gain-of-function polymorphism (e756del) common in African Americans was previously not found to be associated with glaucoma phenotypes^44^. It is unclear if the two alleles we identified confer gain or loss of function. Future studies are needed to elucidate the effects of these alleles on channel function.

Common noncoding SNPs located in the *PIEZO1* and *PIEZO2* genomic regions showed interesting associations with NTG and POAG respectively, although neither of these SNPs had evidence of association in the FinnGen dataset.

Published single-cell and single-nucleus RNA sequencing data^42,43^ shows that *PIEZO1* and *PIEZO2* are expressed in both the ocular anterior segment and the retina, with strongest expression in the lymphatic endothelium, recently recognized as contributing to Schlemm’s canal function^45^. Previous in vitro studies have implicated PIEZO1 and PIEZO2 in aqueous outflow regulation. PIEZO1 activity in trabecular meshwork cells has been proposed to increase conventional aqueous ouflow^32,34,35^. In contrast, modulating PIEZO2 expression in iridocorneal tissues does not affect the conventional outflow pathway or IOP, but may affect unconventional outflow pathways or aqueous humor production^36^. *PIEZO1* and *PIEZO2* are also expressed in astrocytes of the optic nerve head^46^. PIEZO1 has been suggested to contribute to astrocyte reactivity^47^, and PIEZO2 expression in the optic nerve head was upregulated after transient IOP elevation^46^. Future studies are needed to fully elucidate the roles of PIEZO1 and PIEZO2 in different ocular tissues.

Rare missense alleles meeting the threshold for nominal POAG association (*P* < 0.05) in the NEIGHBOR and MEE samples were also found in *TRPA1* (A122V) and in *TMEM63B* (V307M). However, neither of these were associated with disease risk in the UK Biobank or in the FinnGen dataset. The *TMEM63B* variant did show consistent direction of effect in both the UK Biobank and in FinnGen. *TMEM63B* is expressed in retinal ganglion cells (Fig. 2), but the role of these channels in the eye is unknown.

In addition to rare variant association, we also found interesting associations for SNPs located in the genomic regions of *TRPV2*, *TRPM3*, *TRPA1* and *KCNK2* (*TREK1*). A *TRPV2* SNP (rs34419652), an eQTL, was significantly associated with POAG risk in NEIGHBORHOOD and showed a consistent direction of effect in FinnGen. A *TRPM3* SNP (rs3124515) was significantly associated with POAG in NEIGHBORHOOD. A *KCNK2* (*TREK1*) SNP (rs2841593) was associated with POAG risk in the NEIGHBORHOOD dataset, although this result did not replicate in FinnGen. KCNK2 has been suggested to sense mechanical stretch in the trabecular meshwork and regulate IOP^29,30^. The TRP channel genes are expressed in both the anterior segment and the retina (Fig. 2), and have been suggested to play multiple roles in the eye that may contribute to glaucoma risk^48^. TRPV2 contributes to the myogenic constriction of retinal arterioles^49^, and possibly regulates blood flow in the retina. *TRPM3* is expressed in the ciliary body, lens, and retinal pigment epithelium^50,51^, and a missense mutation in *TRPM3* has been linked to congenital cataracts and glaucoma^52^. TRPA1 mediates oxidative stress after retinal ischemia^53^.

Multiple in vitro and animal studies have also suggested roles for TRPV1 and TRPV4 in glaucoma pathogenesis, including roles in IOP regulation^31,37,54,55^, RGC apoptosis and survival^38,40,41^, and regulation of inflammatory cytokines^39^. In this study, we did not find significant associations between *TRPV1* and *TRPV4* SNPs with POAG, although a *TRVP4* SNP (rs12423752) reached threshold significance for NTG in NEIGHBORHOOD.

We selected to investigate a subset of TRP channels that have been implicated in mechanosensation in this study. It remains controversial whether these channels are directly or indirectly activated by mechanical forces^56^. Nonetheless, loss-of-function mutations in humans suggests their involvement in mechanosensory processes^57^.

We have presented an overview of the expression of mechanosensitive genes using previously published human single-cell and single-nucleus RNA sequence data^42,43^ (Fig. 2). Additional histological validation of gene expression would complement this data in elucidating the roles of mechanosensitive channel genes in the eye.

For this gene survey, we used the NEIGHBORHOOD dataset to assess common variation, and two of the NEIGHBORHOOD cohorts (NEIGHBOR and MEE) with exome array data to assess rare coding variation. A strength of our study is that the cases and controls have had clinical examinations. While the sample size is relatively large, it is also potentially limiting for very rare variants and for common variants with small effects. For replication, we used two additional independent datasets: FinnGen, a large population dataset with both common and rare variant association results for a range of glaucoma phenotypes, and the UK Biobank, also a population dataset with glaucoma association results for rare coding variants derived from whole exome sequence data. A limitation of both the FinnGen and the UK Biobank datasets is that glaucoma classification is based on diagnosis codes, and for the UK Biobank also self-report. This may create some variability in glaucoma diagnosis. Another limitation of our analysis is that we used exome array data for assessing rare coding variants. Because the exon array is comprised of selected rare coding variants, it is not equivalent to the full set of variants that can be identified by exome sequencing. Further evaluation of the genes of interest identified in this study using whole exome or whole genome sequencing would be of interest. It would also be valuable to include ethnically diverse populations in future studies.

### Conclusion

We surveyed 4 large mechanosensitive ion channel families—K2P, TMEM63, PIEZO and TRP—to discover associations between genetic variants in these genes and POAG^3,5,6,24^. We find novel variants in several mechanosensitive ion channel genes that show interesting associations with POAG. Future functional studies are warranted to determine the effects of these variants on channel function, as well as on glaucoma phenotypes.

## Methods

### NEIGHBORHOOD study participants

NEIGHBORHOOD case and control recruitment and definitions have been reported previously^58–60^. Briefly, the NEIGHBORHOOD dataset includes eight independent datasets with a total of 3,853 cases and 33,480 controls. A harmonized POAG definition was adopted across these data sets based on the following criteria: (1) open anterior segment angles, (2) reproducible glaucomatous visual field loss on reliable tests or (3) an eye with cup-disc ratio of at least 0.7 with one visual field showing glaucomatous loss, and (4) no identifiable secondary cause for optic nerve disease. Elevated intraocular pressure (IOP) was not a criterion for POAG definition, but if present, there had to be no secondary causes on anterior segment examination.

Sixty-seven percent of cases had a history of elevated IOP (≥ 22 mm Hg) measured in a clinical setting and were classified as HTG (high-tension glaucoma). Cases with IOP < 22 mm Hg (without treatment) measured in the clinic at the time of study enrollment were classified as NTG (normal-tension glaucoma). Cases undergoing IOP-lowering therapy at the time of enrollment were included in the HTG group if they had a documented history of IOP > 22 mm Hg prior to treatment, and cases undergoing IOP-lowering therapy at the time of enrollment were included in the NTG group if they did not have recorded pressures > 22 mm Hg before treatment. Pretreatment IOP measurements were not available for all cases.

### NEIGHBORHOOD GWAS data

Association data for SNPs located within genomic regions that contain the selected mechanosensitive channel genes was extracted from the NEIGHBORHOOD GWAS data^58^. For the GWAS case and control samples were genotyped on Illumina 660W (Illumina, San Diego, CA, USA), Affymetrix 500 K, Affymetrix Mapping 5.0, or Affymetrix 6.0 arrays (Affymetrix, Santa Clara, CA, USA). The genotype imputation was based on 1000 Genomes panel (March 2012). For each dataset, age, sex, and study-specific principal components were adjusted in logistic regression models using ProbABEL^61^. In the meta-analysis, inverse variance weighted method was performed in METAL (2011-03-25 release) with genomic control correction^62^. Association analyses were done for POAG overall as well as the HTG and NTG subgroups^58^.

To examine the NEIGHBORHOOD association results for genomic regions that include the mechanosensitive genes of interest, we extracted data for all SNPs located within the genomic region that included the gene as well as 50 Kb on both the 5’ and 3’ ends of the gene of interest. We did not include TRPC5 because it is located on the X chromosome and the X chromosome was not included in the NEIGHBORHOOD GWAS analysis. The SNP with the smallest *P* value for association was selected as representative of the overall result for each genomic region. We used a p value correction for the number of genomic regions examined to determine statistical significance (*P* = 0.05/20 = 0.0025).

The Gene-Tissue Expression (GTEx) database (https://www.gtexportal.org/home/) was used to identify SNPs that are expression quantitative trait loci (eQTLs) and hence may influence expression levels of the genes of interest.

### Exome array genotyping and association analysis

2606 cases and 2606 controls from the NEIGHBORHOOD NEIGHBOR and MEE cohorts were submitted to the Center for Inherited Disease Research (CIDR) for genotyping using the Illumina HumanExome BeadChip (Illumina, Inc., San Diego, CA). Illumina Genome Studio (Illumina, Inc.) and PLINK^63^ was used for all QC steps, except where noted. Basic QC for samples included screens for call rate (≥ 98.5%) and high (≥ 95%) concordance with previous Illumina 660 K Beadchip genotypes^59^ where available (about 80% of samples). Recorded sex in the clinical records was verified with genotyped sex by two criteria: mean fluorescence intensity on the X and Y chromosomes, plus genotype heterozygosity on the X chromosome and call rate on the Y, allowing male and female samples to have heterozygous X-linked and successful Y-linked genotypes, respectively. Samples were tested for pairwise relationships and unexpected duplication using KING^64^, testing overall genomic sharing by robust kinship estimation, and proportion shared 0, 1 and 2 alleles identical by descent under the assumption of population homogeneity.

European ancestry was verified from the first two principal components derived from genotypes at 9000 ancestry-informative markers by means of the SNPweights program^65^, including representative HapMap CEU, YRI, CHB and JPT samples as reference populations. A principal components analysis was conducted using over 52,040 independent (pairwise *r*^2^ < 0.1), common (MAF ≥ 0.005) SNPs and the smartpca program in EIGENSOFT to detect finer population structure. Of the first 20 principal components, the first, sixth and eighth were significantly associated (*P* < 0.05 by logistic regression) with POAG status.

Initial QC screens for markers included call rate (≥ 98%) and consistency with Hardy–Weinberg proportions (*P* > 10^−6^ by Fisher exact test). Genotype clustering was confirmed for rare (MAF < 0.02) variants using genotype calls from zCall^66^ run with a stringent *Z*-score threshold of *Z* = 21 for calling heterozygous genotypes, from a GenomeStudio report containing genotype calls and X and Y intensity values as input. Every rare SNP with two or more additional heterozygous calls by zCall than by GenCall was reviewed in Genome Studio, and, if necessary, cluster locations were adjusted manually.

Association between single variants and POAG case/control status was tested by logistic regression, including age at exam, sex, and three principal components observed to be significantly associated with POAG as covariates. For the single-variant association test we selected SNPs with minor allele frequencies (MAF) less than 1% and CADD (Combined Annotation Disruption Depletion) scores of > 15. A CADD score of 15 indicates that the variant is in the top 5% of deleterious alleles within the human genome^67^. Gene-based association tests were done using the optimized kernel association test implemented in SKAT-O^68^ for genes with at least 3 variants identified in the exome array data. Covariate adjustments were the same as for the single-variant analysis. We considered the rare variant association exploratory and identified variants of interest as those with *P*-values for association equal to or less than 0.05.

GWAS and exome array association results were compared to association analyses completed using exome data from the UK Biobank^69^ (https://app.genebass.org/) and association analyses using genome-wide genotypes from FinnGen (https://r8.finngen.fi/).

Splice AI (https://spliceailookup.broadinstitute.org/) was used to predict splicing alteration as a consequence of *PIEZO1* canonical donor splice variant (16-88737727-C-G).

### Supplementary Information

Supplementary Tables.

## Data Availability

The summary statistics for the NEIGHBORHOOD genotype data are available at http://eaglep.case.edu/glaucomagenetics_web/ and also through reasonable request. All variants analyzed in this report are included in the manuscript either in tables or as supplemental data.
