# Supplementary material for: Mechanosensitive ion channel gene survey suggests potential roles in primary open angle glaucoma
Source: Sci Rep. 2023 Sep 23;13:15871. doi: 10.1038/s41598-023-43072-3 (PMC10517927; doi:10.1038/s41598-023-43072-3)
Supplement: Supplementary file 1 — Supplementary Tables. [file 41598_2023_43072_MOESM1_ESM.docx]

**Supplemental Information**

Supplemental Table 1. Variants reaching association threshold in NEIGHBHORHOOD and their association results in FinnGen

|  |  | NEIGHBORHOOD | | | FinnGen | | |
| --- | --- | --- | --- | --- | --- | --- | --- |
| Gene | SNP | Glaucoma type | Beta | P | Beta | P | Phenotype |
| KCNK2/TREK1 | rs2841593 | POAG | 0.15 | 0.002 | -0.045 | 0.14 | POAG, strict |
| PIEZO1 | rs12709104 | NTG | -0.37 | 0.002 | 0.014 | 0.77 | Normotensive |
| PIEZO2 | rs112014893 | HTG | 0.29 | 5.40E-04 | -0.032 | 0.36 | POAG, strict |
| TMEM63C | rs8016340 | POAG | -0.11 | 0.002 | 0.0065 | 0.73 | POAG, strict |
| **TRPM3** | rs3124515 | POAG | -0.12 | 0.0013 | not available |  |  |
|  | **rs2026109** | **NTG** | **0.62** | **0.001** | **0.025** | **0.75** | **Normotensive** |
| **TRPV2** | **rs34419652** | **POAG** | **-0.13** | **9.00E-04** | **-0.017** | **0.47** | **POAG, strict** |
|  | rs9901098 | NTG | -0.33 | 0.002 | not available |  |  |
| TRPV4 | rs12423752 | NTG | -0.28 | 6.00E-04 | 0.013 | 0.74 | Normotensive |
| TRPA1 | rs4738210 | HTG | 0.275 | 1.00E-04 | -0.02 | 0.29 | Glaucoma |

SNP = single nucleotide polymorphism

POAG = primary open-angle glaucoma

NTG = normal-tension glaucoma

HTG = high-tension glaucoma

Supplemental Table 2. Gene-based association results for rare variants using SKAT-O

| Gene | NEIGHBORHOOD | UK Biobank | UK Biobank |
| --- | --- | --- | --- |
|  | SKATO P-value | SKATO LoF P-value | SKATO missense P-Value |
| KCNK2/ TREK1 | NA | 0.584 | 0.352 |
| KCNK4/ TRAAK | NA | 0.864 | 0.827 |
| KCNK10/ TREK2 | 0.365 | 0.222 | 0.475 |
| PIEZO1 | NA | 0.808 | 0.763 |
| PIEZO2 | NA | 0.474 | 0.444 |
| TMEM63A | 0.179 | 0.871 | 0.264 |
| TMEM63B | 0.094 | 0.462 | 0.918 |
| TMEM63C | 0.206 | 1 | 0.347 |
| **TRPC1** | NA | 0.719 | **6.34E-03** |
| TRPC3 | 0.87 | 0.806 | 0.842 |
| TRPC5 | NA | 0.125 | 0.837 |
| TRPC6 | 0.345 | 0.162 | 0.851 |
| TRPM3 | 0.327 | 0.833 | 0.207 |
| TRPM4 | 0.494 | 1 | 0.782 |
| TRPM7 | 0.891 | 0.185 | 0.49 |
| TRPV1 | 0.453 | 0.628 | 1 |
| TRPV2 | 0.88 | 0.201 | 0.183 |
| TRPV4 | 0.826 | 0.476 | 1 |
| TRPA1 | 0.13 | 0.73 | 0.78 |
| PKD2/ TRPP1 | 0.384 | 0.765 | 0.144 |

LoF = loss of function

Supplemental Table 3. Single-variant association results for SNPs with minor allele frequencies less than 1% and Combined Annotation Disruption Depletion scores of >15

| Gene | rsID | Chr | Pos GRCh37/hg19 | Alleles | MAF Case | MAF Control | OR | P | Variant | CADD score |
| --- | --- | --- | --- | --- | --- | --- | --- | --- | --- | --- |
| KCNK4/ TRAAK | rs202162247 | 11 | 64060583 | A/C | 0.00124 | 0.00078 | 1.63 | 0.419 | Missense_H31Q | 18.6 |
| PIEZO1 | rs146505418 | 16 | 88801621 | A/G | 0.00141 | 0.00187 | 0.80 | 0.640 | Missense_R531C | 34 |
| PIEZO1 | rs202099525 | 16 | 88788037 | A/G | 0.00124 | 0.00140 | 0.83 | 0.715 | Missense_P1771L | 30 |
| PIEZO1 | rs199752762 | 16 | 88786328 | T/C | 0.00071 | 0.00078 | 0.71 | 0.615 | Missense_V2069M | 28.8 |
| PIEZO1 | rs200555745 | 16 | 88782988 | T/C | 0.00088 | 0.00078 | 1.19 | 0.791 | Missense_R2302H | 28.3 |
| PIEZO1 | rs200970763 | 16 | 88800139 | T/C | 0.00531 | 0.00673 | 0.72 | 0.186 | Missense_G782S | 26.2 |
| PIEZO1 | rs185326407 | 16 | 88793155 | T/C | 0.00441 | 0.00499 | 0.87 | 0.619 | Missense_V1223I | 25.4 |
| PIEZO1 | rs202103485 | 16 | 88800060 | T/C | 0.00530 | 0.00656 | 0.73 | 0.203 | Missense_R808Q | 25 |
| PIEZO1 | rs200029740 | 16 | 88800074 | G/C | 0.00194 | 0.00281 | 0.65 | 0.275 | Missense_Q803H | 23.3 |
| PIEZO1 | rs201442593 | 16 | 88787052 | A/G | 0.00071 | 0.00172 | 0.38 | 0.102 | Missense_R1925W | 23.3 |
| PIEZO1 | rs200031013 | 16 | 88787595 | A/G | 0.00106 | 0.00047 | 1.86 | 0.387 | Missense_R1883W | 23 |
| PIEZO1 | rs191656121 | 16 | 88789709 | T/C | 0.00321 | 0.00287 | 1.42 | 0.308 | Missense_A1455T | 23 |
| PIEZO1 | rs202066744 | 16 | 88790325 | A/T | 0.00089 | 0.00126 | 1.04 | 0.953 | Missense_E1430V | 23 |
| **PIEZO1** | **rs199524784** | **16** | **88804135** | **G/C** | **0.00072** | **0.00241** | **0.38** | **0.0244** | **splice donor** | **22.9** |
| PIEZO1 | rs147153006 | 16 | 88790362 | G/A | 0.00106 | 0.00109 | 0.88 | 0.822 | Missense_Y1418H | 21.4 |
| **PIEZO1** | **rs148870219** | **16** | **88789333** | **T/C** | **0.00053** | **0.00296** | **0.18** | **0.00597** | **Missense_R1527H** | **17.8** |
| PIEZO2 | rs142103035 | 18 | 10675208 | T/G | 0.00053 | 0.00078 | 0.93 | 0.921 | Missense_S2607Y | 27.3 |
| PIEZO2 | rs73946020 | 18 | 10759507 | T/C | 0.00071 | 0.00109 | 0.66 | 0.519 | Missense_V1219M | 22 |
| TMEM63A | rs75188792 | 1 | 226041377 | A/G | 0.00088 | 0.00094 | 0.99 | 0.984 | Missense_R584C | 35 |
| TMEM63B | rs144454929 | 6 | 44107262 | T/C | 0 | 0.000156 | 0.88 | 0.956 | Missense_R156W | 32 |
| TMEM63B | rs146320190 | 6 | 44103073 | A/G | 0 | 0.000156 | 0.24 | 0.533 | Missense_R83Q | 23.5 |
| TMEM63B | rs143307278 | 6 | 44116308 | T/C | 0.000176 | 0.000156 | 1.4 | 0.819 | Missense_R394C | 23.1 |
| **TMEM63B** | **rs4714759** | **6** | **44115169** | **A/G** | **0.1146** | **0.1043** | **1.13** | **0.041** | **Missense_V307M** | **21.7** |
| TMEM63B | rs201065300 | 6 | 44116345 | C/A | 0.000178 | 0.000627 | 0.22 | 0.188 | Missense_N406T | 18.2 |
| TMEM63A | rs115530894 | 1 | 226037778 | C/G | 0.00265 | 0.00483 | 0.57 | 0.079 | Missense_L636V | 25.5 |
| TMEM63C | rs199846522 | 14 | 77706888 | A/G | 0.00300 | 0.00483 | 0.61 | 0.102 | Missense_R334H | 25 |
| TRPC1 | rs78715340 | 3 | 142443450 | A/T | 0.00035 | 0.00078 | 0.45 | 0.344 | Missense_S17T | 20.9 |
| TRPC3 | rs150886370 | 4 | 122835991 | T/C | 0.00123 | 0.00094 | 1.14 | 0.821 | Missense_V356M | 24.1 |
| TRPC3 | rs144632591 | 4 | 122846193 | C/T | 0.00053 | 0.00094 | 0.76 | 0.706 | Missense_I313V | 22.8 |
| TRPC6 | rs117273916 | 11 | 101375528 | A/G | 0.00053 | 0.00094 | 0.84 | 0.805 | Missense_R58W | 27.5 |
| TRPM3 | rs200939544 | 9 | 73255564 | A/G | 0.00071 | 0.00031 | 2.10 | 0.399 | Missense_S300L | 35 |
| TRPM3 | rs139575787 | 9 | 73233790 | A/C | 0.00035 | 0.00062 | 0.95 | 0.950 | Missense_G609V | 27.3 |
| TRPM3 | rs141399885 | 9 | 73152169 | T/C | 0.00564 | 0.00343 | 1.52 | 0.144 | Missense_R1112Q | 26.4 |
| TRPM3 | rs148192709 | 9 | 73151387 | T/C | 0.00159 | 0.00327 | 0.49 | 0.085 | Missense_G1373R | 25.6 |
| TRPM3 | rs117283138 | 9 | 73225639 | G/C | 0.00723 | 0.00655 | 1.00 | 0.991 | Missense_E676D | 16.8 |
| TRPM4 | rs140799936 | 19 | 49703651 | T/A | 0.00088 | 0.00094 | 1.15 | 0.822 | Nonsense_K769X | 54 |
| TRPM4 | rs201907325 | 19 | 49685865 | A/G | 0.00088 | 0.00047 | 2.19 | 0.298 | Missense_A432T | 33 |
| TRPM4 | rs147854826 | 19 | 49703585 | T/C | 0.00194 | 0.00109 | 1.46 | 0.451 | Missense_R747C | 33 |
| TRPM4 | rs150391806 | 19 | 49714497 | T/C | 0.0055 | 0.00422 | 1.21 | 0.475 | Missense_P1059L | 23.8 |
| TRPM4 | rs148763371 | 19 | 49675297 | G/T | 0.00053 | 0.00047 | 1.21 | 0.823 | Missense_L361R | 23.3 |
| TRPM4 | rs146564314 | 19 | 49671952 | A/G | 0.00617 | 0.00639 | 0.93 | 0.748 | Missense_R252H | 23.2 |
| TRPM4 | rs149335121 | 19 | 49671244 | A/G | 0.00018 | 0.00078 | 0.23 | 0.183 | Missense_R113H | 23.2 |
| TRPM4 | rs172149856 | 19 | 49691898 | A/G | 0.00088 | 0.00047 | 2.19 | 0.298 | Missense_G582S | 20.3 |
| TRPM7 | rs35648842 | 15 | 50899447 | T/A | 0.00106 | 0.00047 | 2.38 | 0.231 | Missense_W887R | 28.7 |
| TRPM7 | rs199732064 | 15 | 50884348 | A/G | 0.00071 | 0.00094 | 0.75 | 0.667 | Missense_P1362S | 23.4 |
| TRPM7 | rs56288221 | 15 | 50884671 | A/G | 0.00088 | 0.00078 | 1.01 | 0.986 | Missense_A1254V | 22.8 |
| TRPV1 | rs200601093 | 17 | 3477184 | G/A | 0.00035 | 0.00078 | 0.49 | 0.405 | Missense_W616R | 17.9 |
| TRPV1 | rs200426339 | 17 | 3470230 | A/G | 0.00071 | 0.00031 | 2.05 | 0.410 | Missense_A800V | 17.0 |
| TRPV4 | rs55728855 | 12 | 110221524 | T/C | 0.00970 | 0.00888 | 1.15 | 0.475 | Missense_E733K | 26.8 |
| TRPV4 | rs116035946 | 12 | 110221544 | C/T | 0.00247 | 0.00249 | 0.95 | 0.896 | Missense_N726S | 22.3 |
| TRPV4 | rs56177950 | 12 | 110230597 | T/C | 0.00882 | 0.00951 | 0.90 | 0.603 | Missense_V455I | 19.2 |
| **TRPA1** | **rs61758122** | **8** | **72981337** | **A/G** | **0.00176** | **0.00047** | **4.58** | **0.024** | **Missense_A122V** | **25** |
| TRPA1 | rs61753709 | 8 | 72963040 | T/C | 0.00388 | 0.00436 | 0.96 | 0.886 | Missense_M626I | 23 |
| PKD2/ TRPP1 | rs147654263 | 4 | 88989111 | A/G | 0.00265 | 0.00327 | 0.89 | 0.743 | Missense_R807Q | 29.1 |
| PKD2/ TRPP1 | rs2234917 | 4 | 88989089 | C/A | 0.00476 | 0.00390 | 0.88 | 0.654 | Missense_M800L | 18.8 |

MAF = minor allele frequency

CADD = Combined annotation dependent depletion

OR = odds ratio
